# Study on the association between the polymorphism of MCP-1 rs1024611 and the genetic susceptibility of type 2 diabetes with sepsis

**DOI:** 10.1097/MD.0000000000029903

**Published:** 2022-08-12

**Authors:** Yan Li, Junbing He, Yi-ming Shao, Lanchun Chen, Ming Li, Donghui Tang, Zhizhou Shi, Qinghui Liao, Zhongqiu Guo, Juan Wang, Qiaoan Zheng, Yanni Zhao, Yuhua Chen

**Affiliations:** a Department of Endocrinology and Metabolism, The Second Affiliated Hospital,School of Medicine, The Chinese University of Hong Kong, Shenzhen, Shenzhen, Guangdong, People’s Republic of China.; b Department of Critical Care Medicine,Affiliated Hospital of Guangdong Medical University, Zhanjiang, Guangdong, People’s Republic of China.; c Department of Critical Care Medicine,The Second Affiliated Hospital,School of Medicine,The Chinese University of Hong Kong,Shenzhen, Shenzhen, Guangdong, People’s Republic of China.; d Department of Physical Examination,The Second Affiliated Hospital,School of Medicine,The Chinese University of Hong Kong,Shenzhen, Shenzhen, Guangdong, People’s Republic of China.; e Department of Gynecology and Obstetrics, Kuichong People’s Hospital, Shenzhen, Guangdong, People’s Republic of China.; f Department of Nutrition,The Second Affiliated Hospital,School of Medicine,The Chinese University of Hong Kong,Shenzhen, Shenzhen, Guangdong, People’s Republic of China.; g Department of Endocrinology, Zhanjiang Central People’s Hospital, Zhangjiang, People’s Republic of China.

**Keywords:** MCP-1, polymorphism, sepsis, type 2 diabetes mellitus

## Abstract

Monocyte chemoattractant protein-1 (MCP-1) rs1024611 (-2518 A > G) polymorphism are associated with inflammatory diseases. In this study, we investigate the relationship between MCP-1 rs1024611 polymorphism and genetic susceptibility of type 2 diabetes mellitus (T2DM) with sepsis.

Two hundred eighty-five patients with T2DM are divided into the diabetes with sepsis group (combined group, 113 cases) and the diabetes group (172 cases). Blood samples and corresponding clinical data were collected. MCP-1 rs1024611 polymorphism in blood samples was detected by pyrosequencing. Meanwhile, the expressions of MCP-1, tumor necrosis factor-alpha (TNF-α), interleukin (IL)-1β, and IL-6 in blood samples were detected by real-time quantitative polymerase chain reaction and enzyme-linked immunosorbent assay, respectively. The relationship between different genotypes of MCP-1 rs1024611 polymorphic locus and T2DM with sepsis was analyzed by combining with the clinical data of the patients.

The frequencies of rs1024611 AG/GG genotypes and G allele in T2DM with sepsis group were significantly higher than those in T2DM patients without sepsis (*P* = .004 for AG/GG vs AA genotypes; *P* = .037 for G allele vs A allele). Subgroup analysis showed that the rs1024611 G allele frequency in the septic shock group was significantly higher than the general sepsis group (*P* = .02). The expressions of MCP-1 and TNF-α in GG genotypes in T2DM with sepsis group were significantly higher than AA or GA genotypes (*P* < .05).

This study preliminarily showed that the rs1024611 A > G polymorphism within the promoter region of MCP-1 gene can upregulate the expression of MCP-1 gene and proinflammatory cytokine TNF-α, which ultimately contributed to the predisposition and progression of T2DM with sepsis.

## 1. Introduction

Type 2 diabetes mellitus (T2DM), a group of metabolic diseases characterized by insulin resistance and hyperglycemia, is caused by progressive failure of pancreatic islet B cell function. Diabetes-associated infection is a common complication of diabetes and an important cause of death from diabetes.^[1,2]^ Sepsis is a complex clinical syndrome caused by the interaction between the infectious pathogen and the host immune system, inflammatory response, and blood coagulation response, which causes damage to multiple organ functions.^[3]^ It is reported that about 17% of patients with sepsis have DM,^[4]^ which may be an important coexisting disease of sepsis.

It has been demonstrated that diabetic patients exhibited an increased risk of developing infection in sepsis and constituted 20.1% to 22.7% of all sepsis patients.^[5]^ Increased rates of colonization by resistant pathogens (e.g., methicillin-resistant *Staphylococcus aureus*) were found in diabetic patients than nondiabetics.^[6]^ Furthermore, sepsis patients with DM possessed more worse deformability of red blood cells, microcirculation, and organ dysfunction than sepsis patients without DM.^[7,8]^ Importantly, several lines of studies showed that having DM worsened clinical prognosis of septic patients, as presented by higher sepsis mortality.^[9]^ Monocyte chemoattractant protein-1 (MCP-1), also known as CC motif chemokine ligand 2, is a member of the CC subfamily (also known as β subfamily) of chemokines, which is mainly chemotactic on the cell surface. The combination of factor receptor 4 (CCR4) and CCR2 activates the signal transduction pathway and plays an important role in the pathophysiological mechanism of diabetes and sepsis.^[10–12]^

The human MCP-1 gene with a genomic region of 79 kb is located on chromosome 17q11.2-q12, which encodes a protein of 76 amino acids. Several studies have shown rs2857656 and rs4586 as MCP-1 functional polymorphisms to affect MCP-1 expression, leading to the susceptibility to pulmonary tuberculosis, spinal tuberculosis, and chronic obstructive pulmonary disease.^[13–15]^ Another functional genetic polymorphism at rs1024611 (-2518 A > G) in the promoter region of MCP-1 gene affects the expression level of MCP-1 and is associated with a variety of inflammatory diseases. MCP-1 rs1024611 (-2518 A > G) polymorphism can affect the transcriptional activity of MCP-1 and is related to the susceptibility of diabetic foot ulcers, inflammatory bowel disease, and sepsis.^[10,15–17]^ However, the relationship between MCP-1 gene polymorphism and diabetes with sepsis is still unclear. This study aimed to analyze the correlation between MCP-1 rs1024611 (-2518 A > G) gene polymorphism and the onset and development of T2DM with sepsis.

## 2. Methods

### 2.1. Objects and groups

In this study, a hospital-based case–control study method was used to enroll 285 subjects. Observation group: in accordance with the 2016 ADA Diabetes Medical Diagnosis and Treatment Standards^[18]^ and the 2016 International Sepsis Guidelines,^[19]^ 113 patients with T2DM and sepsis who were admitted to Longgang District People’s Hospital of Shenzhen and The Third Affiliated Hospital (Provisional) of The Chinese University of Hong Kong and the ICU of Guangdong Medical University Affiliated Hospital between March 2017 and March 2019 were included. Control group: 172 patients simply with T2DM who were admitted to the Endocrinology Department of Shenzhen Longgang District People’s Hospital and Guangdong Medical University Affiliated Hospital during the same period. Exclusion criteria: combined tumors, autoimmune diseases, and blood diseases. General clinical data of the subjects including gender, age, infection site, infectious pathogens, and Acute Physiology and Chronic Health Evaluation II score were collected. This study has been approved by the ethics committee of Longgang District People’s Hospital of Shenzhen, and the subjects and family members have signed an informed consent form. According to the 2016 International Sepsis Guidelines Sepsis 3.0 Standard, the sepsis group is divided into 2 subgroups: general sepsis group (67 cases) and septic shock group (46 cases).

### 2.2. Detection of the genotype of the polymorphic site of the target gene

In this study, the SNaPshot method (Shanghai Tianhao Biotechnology Co., Ltd) was used to detect the genotype of the sample MCP-1 rs1024611. The blood genomic DNA was extracted using a whole blood DNA kit (TIANamp), and the purity of the extracted DNA was tested with gel electrophoresis and Epoch spectrophotometer. The ABI 3130xl DNA gene sequence analyzer (ABI, CA) was used to detect the genotype information of the MCP-1 gene polymorphism site. The primer sequences are as follows: rs1024611F, 5\u697′-CTCTCACGCCAGCACTGACCTC-3\u697′; rs1024611R, 5\u697′-CCAATTAGCCCATGGTCACAGA-3\u697′. Information analysis was performed with GeneMapper 4.1 (Applied Biosystems Co., Ltd, USA) analysis software, and the genotype at the rs1024611 locus of the MCP-1 gene was obtained.

### 2.3. Real-time fluorescence quantitative polymerase chain reaction method to detect target gene expression level

Total RNA was extracted from peripheral blood single cells. Human MCP-1, GAPDH primers were designed and synthesized by Shanghai Shenggong Biological Company. Reverse translation of RNA to cDNA was performed by using the RevertAid™ First Strand cDNA Synthesis Kit (Termo Fisher Scientific). Then SYBR Green mix (Takara) was used for quantitative real-time polymerase chain reaction reaction in an ABI7500 real-time polymerase chain reaction system (Applied Biosystems). GAPDH was used as an internal reference, and the 2^–ΔΔCT^ method was adapted to analyze gene expression differences. The detected primer sequences are as follows: MCP-1 upstream primer 5\u697′-CTCGCCTCCAGCATGAAAGT-3\u697′, downstream primer 5\u697′-GGTGACTGGGGCATTGATTG-3\u697′; GAPDH upstream primer 5\u697′-CTGACTTCAACAGCGACACC-3\u697′; downstream primer 5\u697′-GTGGTCCAGGGGTCTTACTC-3\u697′.

### 2.4. Enzyme-linked immunosorbent assay detects the expression levels of TNF-α, IL-1β, and IL-6 in blood samples

The levels of tumor necrosis factor-alpha (TNF-α), interleukin-1 beta (IL-1β), and interleukin-6 (IL-6) in plasma were detected with enzyme-linked immunosorbent assay kit (Boster Biological Technology Co., Ltd, Wuhan, China) using the double antibody sandwich method according to the instructions. The minimum detectable concentrations of TNF-α, IL-1β, and IL-6 were all 1 pg/mL. Combined with rs1024611 genotyping data, the blood levels of TNF-α, IL-6, and IL-1β in individuals with different genotypes were compared, and the test results were statistically analyzed.

### 2.5. Statistical analyses

The experimental data were statistically analyzed using SPSS Version 20.0 software. The Hardy–Weinberg equilibrium was performed to detect the deviation of genotype/allele frequency in the control and sepsis groups. Power analysis with QUANTO 1.2 software showed 98.0% power for rs1024611 to detect a relative risk difference between genotypes at the significance level of 0.05 and an odds ratio of 2.0 according to our sample size. The chi-square or Fisher exact test was used to analyze the association between MCP-1 polymorphism and type 2 diabetes with sepsis, then we used Benjamin–Hochberg procedure for this multiple-testing correction to analyze the false discovery rate. Student *t* test or Mann–Whitney *U* test was performed to detect the difference between the means of 2 independent samples. The effects of diagnosis and MCP-1 variant on expression of MCP-1 gene and proinflammatory cytokines (TNF-α, IL-6, and IL-1β) were analyzed statistically by 2-way analysis of variance with post hoc Bonferroni correction of group means. A *P* value of <.05 was considered statistically significant.

## 3. Results

### 3.1. General clinical data of the included subjects

The basic clinical data of the subjects (113 cases in the T2DM combined with sepsis group and 172 cases in the T2DM group) are shown in Table 1. There was no statistical difference in age and gender distribution between the 2 groups. In the T2DM with sepsis group, the main source of infection was the respiratory tract (63.7%), gastrointestinal tract (16.8%), and bloodstream infection (15.0%), and the dominating pathogens are *Acinetobacter baumannii* (24.8%), *Klebsiella pneumoniae* (9.7%), and *Staphylococcus aureus* (8.8%); according to the sepsis 3.0 diagnostic criteria, the sepsis group was further divided into general sepsis group (67 cases) and septic shock group (46 cases). The 28-day ICU mortality rate was 29.2%.

**Table 1:** Clinical data of case group and control group.

**Variable**	**T2DM and Sepsis (n = 113) N (%**)	**Control (n = 172) N (%**)	***P* value**
Demographics			
Age (yr), mean ± SD	59.37 ± 16.1	56.56 ± 13.5	.101
Number (male/female)	78/35	122/50	.731
Sepsis status, n (%)			
Mild sepsis	67 (59.3)	NA	
Septic shock	46 (40.7)	NA	
Source of infection, n (%)			
Respiratory tract infection	72 (63.7)	NA	
Primary bloodstream infection	17 (15.0)	NA	
Abdominal infection	19 (16.8)	NA	
Urinary tract infection	10 (8.8)	NA	
Catheter-associated infection	6 (5.3)	NA	
Central nervous system infections	9 (8.0)	NA	
Others	14 (12.4)	NA	
Infection types, n (%)			
*Acinetobacter baumannii*	28 (24.8)	NA	
*Monilia albican*	9 (8.0)	NA	
Yeast sample sporphyte	6 (5.3)	NA	
*Aspergillus*	4 (3.5)	NA	
*Klebsiella pneumoniae*	11 (9.7)	NA	
*Pseudomonas aeruginosa*	9 (8.0)	NA	
*Staphylococcus aureus*	10 (8.8)	NA	
*Escherichia coli*	13 (11.5)	NA	
Others	19 (16.8)	NA	
qSOFA score, mean ± SD	2.53 ± 0.50	NA	
Respiratory rate ≥22/min, n (%)	103 (91.2)	NA	
Altered mentation, n (%)	85 (75.2)	NA	
Systolic blood pressure ≤100 mm Hg, n (%)	98 (86.7)	NA	
SOFA score, mean ± SD	8.65 ± 4.68	NA	
APACHE II score, mean ± SD	24.3 ± 6.8	NA	
28-day mortality, n (%)	33 (29.2)	NA	

### 3.2. The relationship between the polymorphism of MCP-1 rs1024611 and the susceptibility of T2DM with sepsis

The frequency distribution of rs1024611 genotype and alleles in the T2DM combined with the sepsis group and the T2DM control group are shown in Table 2. No significant deviation from Hardy–Weinberg equilibrium was detected for rs1024611 in both the sepsis and control groups (both *P* > .05). The genotype distributions between 2 groups were significantly different (*P* = .015). Compared with the control group, the frequency of rs1024611 AG/GG genotype in the T2DM combined with sepsis group was significantly higher than that in the control group (*P* = .004: AG + GG vs AA). The frequency of the rs1024611G allele was significantly higher in the diabetic combined with sepsis group than in the T2DM control group (*P* = .037).

**Table 2:** Distribution of genotype and allele frequency in case group and control group.

**MCP-1**	**T2DM with sepsis, n = 113 (%**)	**Control, n = 172 (%**)	***P* value**	***P* value** *	**Odds ratio (95% CI**)
Additive model					
AA	19 (16.8)	55 (32.0)	–	–	1.000 (reference)
AG	64 (56.6)	76 (44.2)	.005	.015	2.438 (1.313–4.525)
GG	30 (26.5)	41 (23.8)	.036	.056	2.118 (1.049–4.277)
Additive model					
AA	19 (16.8)	55 (32.0)	.036	.056	0.472 (0.234–0.953)
AG	64 (56.6)	76 (44.2)	.633	.633	1.151 (0.647–2.048)
GG	30 (26.5)	41 (23.8)	–	–	1.000 (reference)
Dominant model AA/AG vs GG	83 (73.5)	131 (76.2)	.605	.633	1.155 (0.669–1.993)
Recessive model AA vs AG/GG	94 (83.2)	117 (68.0)	.004	.015	0.430 (0.239–0.774)
A allele	102 (45.1)	186 (54.1)	–	–	1.000 (reference)
G allele	124 (54.9)	158 (45.9)	.037	.056	1.431 (1.022–2.005)
HWE, P	0.127	0.148	–	–	–

### 3.3. Differences in the distribution of MCP-1 rs1024611 allele and genotype frequencies in different sepsis subgroups

Based on the severity of sepsis, we further divided 113 patients with T2DM with sepsis into the general sepsis group and septic shock group to assess the potential relationship between MCP-1 gene polymorphism and sepsis progression. As shown in the subgroup analysis in Table 3, the frequency of the rs1024611G allele in the T2DM with septic shock group was significantly higher than that in the T2DM with general sepsis group (*P* = .02), revealing that the rs1024611G allele may have a potential role in the progression of T2DM with sepsis from general sepsis to septic shock. Besides, T2DM patients with sepsis carrying GA/GG genotypes exhibited significantly higher Acute Physiology and Chronic Health Evaluation II score than those with AA genotype (*P* < .05).

**Table 3:** Genotype and allele frequency distribution of MCP-1 in different subgroups of sepsis with T2DM.

**MCP-1**	**General sepsis, n = 67 (%**)	**Septic shock, n = 46 (%**)	***P* value**	***P* value** *	**Odds ratio (95% CI**)
Additive model					
AA	15 (22.4)	4 (8.7)	–	–	1.000 (reference)
AG	39 (58.2)	25 (54.3)	.156	.156	2.404 (0.715–8.076)
GG	13 (19.4)	17 (37.0)	.018	.060	4.904 (1.312–18.326)
Additive model					
AA	15 (22.4)	4 (8.7)	.018	.060	0.204 (0.055–0.762)
AG	39 (58.2)	25 (54.3)	.112	.134	0.490 (0.203–1.181)
GG	13 (19.4)	17 (37.0)	–	–	1.000 (reference)
Dominant model AA/AG vs GG	54 (80.6)	29 (63.0)	.051	.102	2.435 (1.053–5.461)
Recessive model AA vs AG/GG	15 (22.4)	4 (8.7)	.074	.111	0.330 (0.113–1.066)
A allele	69 (51.5)	33 (35.9)	–	–	1.000 (reference)
G allele	65 (48.5)	59 (64.1)	.020	.060	1.898 (1.101–3.271)

### 3.4. The relationship between MCP-1 rs1024611 polymorphism and MCP-1 expression level

**Figure 1.**
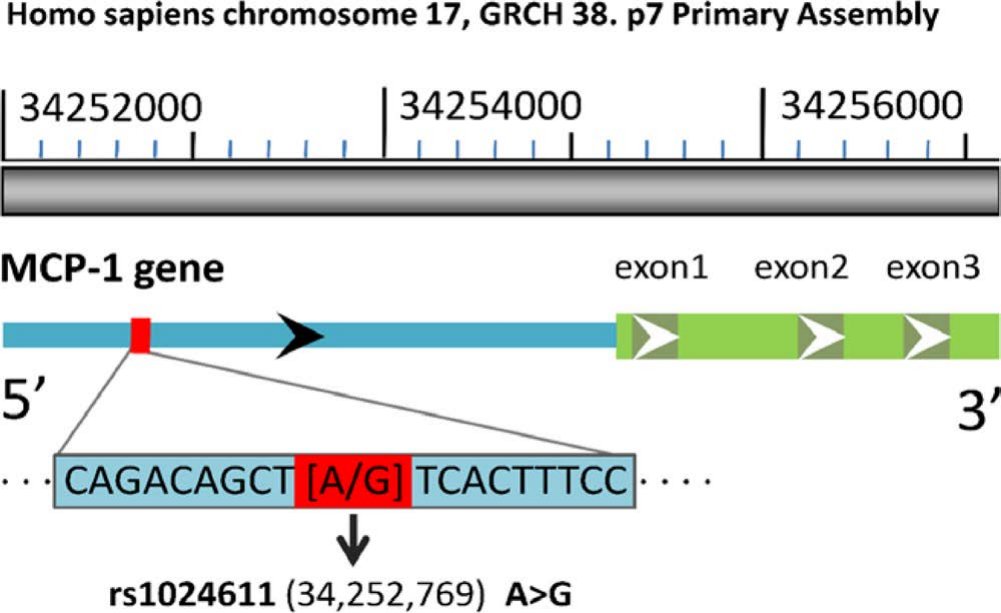
The promoter region of MCP-1 gene and the location of rs1024611 polymorphism. Human MCP-1 gene is located on human chromosome 17 (34,255,277–34,257,203). The blue bar represents the 5′ UTR of MCP-1 gene, and 3 dark green bars represent 3 exons, respectively .rs1024611 is located upstream of the transcription initiation site (-2518 BP). MCP-1 = monocyte chemoattractant protein-1, UTR = untranslated region.

**Figure 2.**
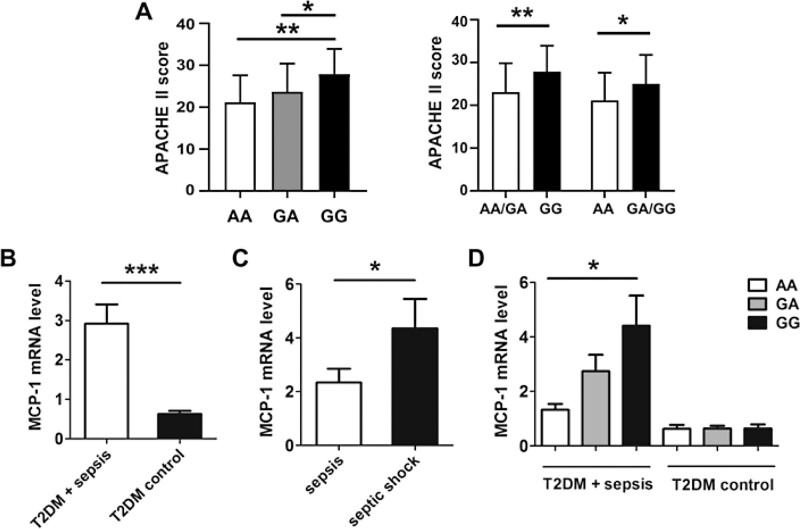
APACHE II score and MCP-1 expression in sepsis patients with T2DM. APACHE II score in sepsis patients with T2DM according to different genotypes of rs1024611 (A); MCP-1 mRNA expression in blood samples of case group (24 cases) and control group (36 cases). MCP-1 mRNA expression level (B) in T2DM with sepsis group and control group, MCP-1 mRNA expression level in T2DM with general sepsis group and septic shock group (C), plasma MCP-1 expression in T2DM and sepsis with different rs1024611 genotype (D). (*P < .05; **P < .01; ***P < .001). APACHE II = Acute Physiology and Chronic Health Evaluation. MCP-1 = monocyte chemoattractant protein-1, T2DM = type 2 diabetes mellitus.

The location of the rs1024611 polymorphism in the promoter region of the MCP-1 gene is presented in Figure 1. We then randomly selected 24 patients with T2DM combined with sepsis and 36 patients with T2DM in the control group to detect the expression of MCP-1 mRNA in peripheral blood mononuclear cells. As shown in Figure 2, the expression level of MCP-1 mRNA in the T2DM combined with sepsis group was significantly higher than that in the control group (*P* < .001). In the sepsis subgroup, the expression of MCP-1 mRNA in the septic shock group was significantly higher than that in the general sepsis group (*P* < .05). In addition, we further evaluated the effects of different genotypes of MCP-1 rs1024611 on the expression of MCP-1 mRNA and found that the expression of GG genotype in the T2DM combined with the sepsis group was significantly higher than the AA and GA genotypes (*P* < .05). However, there was no significant difference in MCP-1 mRNA expression of the 3 genotypes in the control group (*P* < .05).

### 3.5. The relationship between MCP-1 rs1024611 polymorphism and the expression of inflammatory factors

**Figure 3.**
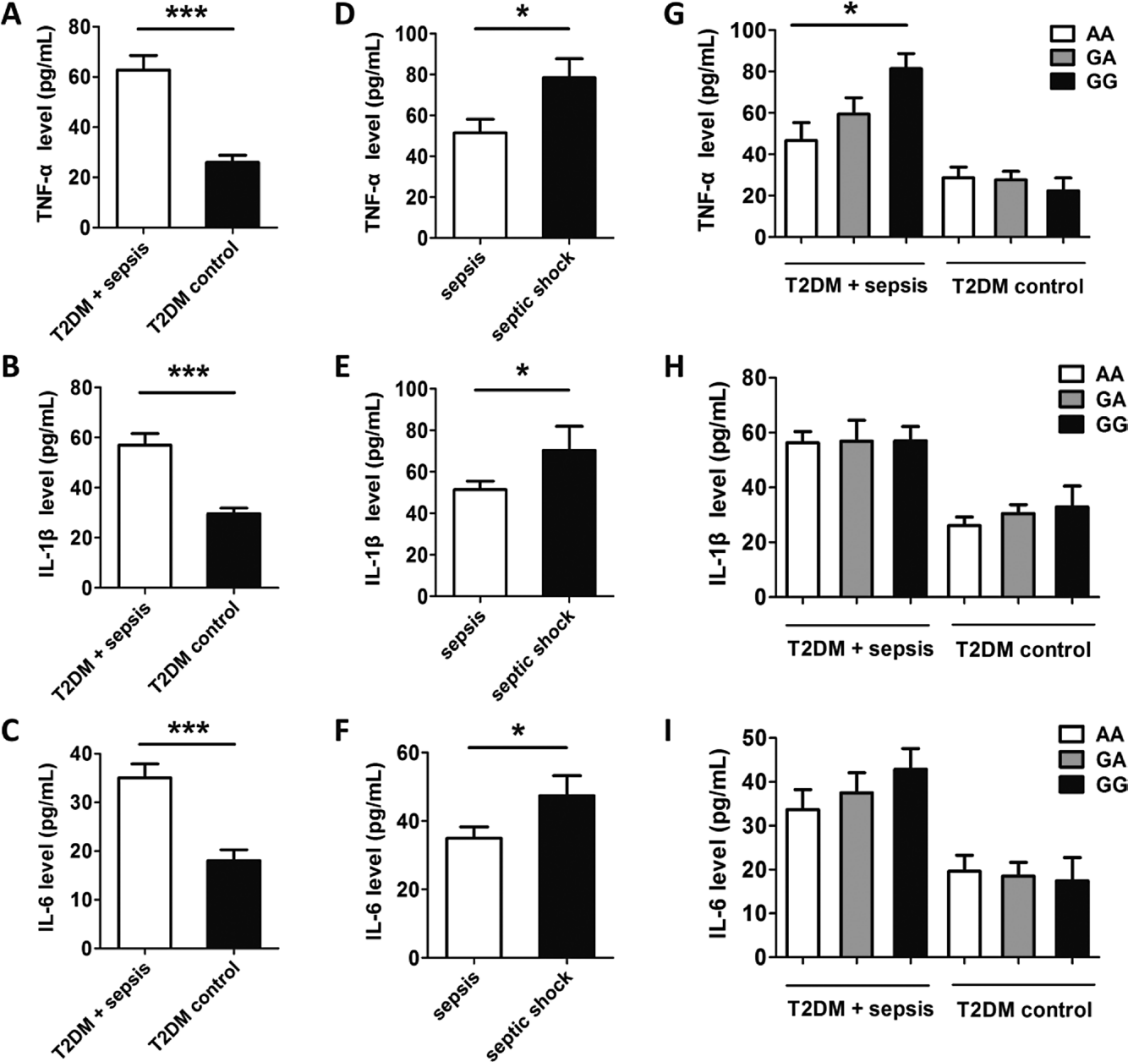
The concentration of inflammatory factors in peripheral blood of 24 diabetes patients complicated with sepsis and control groups of 36 patients with T2DM. The expression levels of TNF-α (A), IL-6 (B), IL-1β (C) in T2DM are complicated with sepsis and control group. The expression levels of TNF-α (D), IL-6 (E) and IL-1β (F) in T2DM with general sepsis group and septic shock group. And the expression of TNF-α (G), IL-6 (H) and IL-1β (I) in T2DM with sepsis of different rs1024611 genotypes. (*P < .05; **P < .01; ***P < .001). IL-1β = interleukin-1 beta, IL-6 = interleukin-6, MCP-1 = monocyte chemoattractant protein-1, T2DM = type 2 diabetes mellitus, TNF-α = tumor necrosis factor-alpha.

To further determine whether the MCP-1 rs1024611 polymorphism will affect the production of these inflammatory factors in these groups, we measured the plasma TNF-α, IL-6, and IL-1β concentration. As shown in Figure 3, the plasma levels of TNF-α, IL-6, and IL-1β in the T2DM combined with the sepsis group were significantly higher than those in the control group (*P* < .001). In the sepsis subgroup, the expression levels of inflammatory factors in the septic shock group were significantly higher than those in the general sepsis group (*P* < .05). In addition, compared with the rs1024611AA/GA genotype, the TNF-α concentration in the carriers of the MCP-1 rs1024611GG genotype was significantly higher than that of the rs1024611AA/GA genotype (*P* < .05). However, the concentration of IL-6 and IL-1β of different genotypes was similar in the T2DM combined with sepsis group and control group.

## 4. Discussion

A number of studies indicate that genetic variation plays an important role in the occurrence and development of diseases,^[20,21]^ in which MCP-1 single nucleotide polymorphism (SNP) has been widely reported to be related to T2DM, sepsis, and other inflammatory conditions.^[22]^ In this study, we compared the allele frequency and genotype frequency of MCP-1 rs1024611 between the T2DM with sepsis group and the T2DM control in a case–control association analysis. The relationship between the rs1024611 polymorphism in the MCP-1 promoter region and the occurrence and development of T2DM with sepsis. The results showed that the ratio of rs1024611 AG/GG genotype frequency and G allele frequency in the T2DM with sepsis group was significantly higher than that in the control group. The ratio of rs1024611G allele frequency in the septic shock group was significantly higher than that of the general sepsis group. Our data suggest that MCP-1 SNP may serve as a risk factor for the occurrence and development of T2DM with sepsis.

The human MCP-1 gene located on chromosome 17q11.2-q12. MCP-1 plays a key role in regulating monocyte chemotaxis and endothelial activation, as well as regulating the progression of inflammation and the production of proinflammatory cytokines.^[23]^ Lee et al^[24]^ have reported that the MCP-1 rs1024611 polymorphism is associated with Alzheimer disease,^[24]^ asthma,^[25]^ immunoglobulin A nephropathy,^[26]^ lupus nephritis,^[27]^ diabetic nephropathy,^[28]^ and diabetic foot ulcer. Inhibition of MCP-1 or specific MCP-1 antagonist can inhibit the release of TNF-α, IL-1β, and IL-6 from macrophages.^[29]^ In this study, we found that the plasma levels of TNF-α, IL-1β, and IL-6 in the T2DM combined with sepsis group were significantly higher than those in the control group, and the levels of these cytokines also increased with the severity of sepsis. More importantly, we observed that the plasma TNF-α in patients with T2DM and sepsis carrying the rs1024611 GG genotype was upregulated with MCP-1. Although IL-1β and IL-6 did not increase simultaneously with MCP-1 as expected, this may be due to the small number of cases and the complex regulation of inflammatory response in the body. We infer that the rs1024611 G allele may increase the transcriptional activity of MCP-1 gene and upregulate the expression level of MCP-1, leading to overactivation of macrophages, increasing the production of proinflammatory cytokines, and ultimately leading to T2DM with sepsis. The detailed mechanism of the occurrence and development of the disease remains to be further studied.

There are certain limitations to this study. First, the small number of research subjects may affect our preliminary conclusions. Second, we only explored the relationship between the polymorphism of a single locus of the MCP-1 gene and T2DM with sepsis. The association of other functional polymorphisms with T2DM with sepsis needs to be further identified. Therefore, in the future, it is still necessary to increase the sample size of the study subjects and increase the detection of other functional polymorphisms of MCP-1 to verify the relationship between MCP-1 polymorphism and T2DM with sepsis.

## 5. Conclusions

In summary, this study proved for the first time that the MCP-1 gene polymorphism rs1024611 G allele/GG haplotype is related to the susceptibility and protection of T2DM with sepsis. The high-risk genotype GG of rs1024611 affects the transcriptional activity and expression of MCP-1. These findings reveal the important clinical significance of the MCP-1 polymorphism and also verify the importance of MCP-1rs1024611 G in the occurrence and development of T2DM with sepsis.

## Author contributions

Conceptualization: Yan Li, Junbing He, Yi-ming Shao, Yuhua Chen

Data curation: Yan Li, Junbing He

Formal analysis: Yan Li, Junbing He

Funding acquisition: Yi-ming Shao, Yuhua Chen

Investigation: Yan Li, Junbing He, Lanchun Chen, Ming Li, Donghui Tang, Zhizhou Shi, Qinghui Liao, Zhongqiu Guo, Juan

Wang, Qiaoan Zheng, Yanni Zhao

Methodology: Lanchun Chen, Ming Li, Donghui Tang, Zhizhou Shi, Qinghui Liao, Zhongqiu Guo, Juan Wang, Qiaoan Zheng, Yanni Zhao,

Project administration: Yi-ming Shao, Yuhua Chen

Resources: Yi-ming Shao, Yuhua Chen

Software: Yan Li, Junbing He

Supervision: Yi-ming Shao, Yuhua Chen

Validation: Yan Li, Junbing He

Visualization: Yan Li, Junbing He, Lanchun Chen, Ming Li, Donghui Tang, Zhizhou Shi, Qinghui Liao, Zhongqiu Guo, Juan

Wang, Qiaoan Zheng, Yanni Zhao

Writing – original draft: Yan Li, Junbing He

Writing – review & editing: Yan Li, Junbing He, Yi-ming Shao, Yuhua Chen

## Footnotes

* False discovery rate-adjusted *P* value for multiple hypotheses testing using the Benjamin–Hochberg method.

* False discovery rate-adjusted *P* value for multiple hypotheses testing using the Benjamin–Hochberg method.
